# Prognostic potential of automated Ki67 evaluation in breast cancer: different hot spot definitions versus true global score

**DOI:** 10.1007/s10549-020-05752-w

**Published:** 2020-06-22

**Authors:** Stephanie Robertson, Balazs Acs, Michael Lippert, Johan Hartman

**Affiliations:** 1 Department of Oncology and Pathology, CCK, Karolinska Institutet, R8:04, 17176 Stockholm, Sweden; 2 Department of Clinical Pathology and Cytology, Karolinska University Laboratory, Stockholm, Sweden; 3 Visiopharm A/S, Hoersholm, Denmark

**Keywords:** Breast cancer, Ki67, Digital pathology, Digital image analysis, Immunohistochemistry

## Abstract

**Purpose:**

The proliferation-associated biomarker Ki67 has potential utility in breast cancer, including aiding decisions based on prognosis, but has unacceptable inter- and intralaboratory variability. The aim of this study was to compare the prognostic potential for Ki67 hot spot scoring and global scoring using different digital image analysis (DIA) platforms.

**Methods:**

An ER+/HER2− breast cancer cohort (*n* = 139) with whole slide images of sequential sections stained for hematoxylin–eosin, pancytokeratin and Ki67, was analyzed using two DIA platforms. For hot spot analysis virtual dual staining was applied, aligning pancytokeratin and Ki67 images and 22 hot spot algorithms with different features were designed. For global Ki67 scoring an automated QuPath algorithm was applied on Ki67-stained whole slide images. Clinicopathological data included overall survival (OS) and recurrence-free survival (RFS) along with PAM50 molecular subtypes.

**Results:**

We show significant variations in Ki67 hot spot scoring depending on number of included tumor cells, hot spot size, shape and location. The higher the number of scored tumor cells, the higher the reproducibility of Ki67 proliferation values. Hot spot scoring had greater prognostic potential for RFS in high versus low Ki67 subgroups (hazard ratio (HR) 6.88, CI 2.07–22.87, *p* = 0.002), compared to global scoring (HR 3.13, CI 1.41–6.96, *p* = 0.005). Regarding OS, global scoring (HR 7.46, CI 2.46–22.58, *p* < 0.001) was slightly better than hot spot scoring (HR 6.93, CI 1.61–29.91, *p* = 0.009). In adjusted multivariate analysis, only global scoring was an independent prognostic marker for both RFS and OS. In addition, global Ki67-based surrogate subtypes reached higher concordance with PAM50 molecular subtype for luminal A and B tumors (66.3% concordance rate, *κ* = 0.345), than using hot spot scoring (55.8% concordance rate, *κ* = 0.250).

**Conclusions:**

We conclude that the automated global Ki67 scoring is feasible and shows clinical validity, which, however, needs to be confirmed in a larger cohort before clinical implementation.

**Electronic supplementary material:**

The online version of this article (10.1007/s10549-020-05752-w) contains supplementary material, which is available to authorized users.

## Introduction

Tumor proliferation is one of the hallmarks of cancer. The proliferation-associated nuclear protein Ki67 is expressed in all phases of the cell cycles except for G_0_ [1]. In many countries, immunohistochemistry-based assessment of Ki67 is part of the routine biomarker evaluation of breast cancers along with estrogen receptor (ER), progesterone receptor (PR) and human epidermal growth factor receptor 2 (HER2). Ki67 has been used for over two decades as a prognostic biomarker in early breast cancer [2–4], and tumor proliferation may be used to guide clinical decisions concerning chemotherapy [5].

Breast cancer is a heterogeneous disease and can be classified into the intrinsic molecular subtypes: luminal A, luminal B, HER2-enriched and basal-like [6]. These intrinsic subtypes as first described by Sorlie and Perou hold both predictive and prognostic information [7, 8]. The majority of luminal tumors are hormone receptor (HR)-positive and account for 70% of all breast cancer cases. Luminal A tumors have low proliferation and good prognosis with high sensitivity to endocrine therapy [9, 10], whereas luminal B tumors are highly proliferative and are less sensitive to endocrine therapy with a poorer prognosis [11, 12]. The HER2-enriched subtypes are aggressive tumors with poor prognosis; however, they are effectively targeted by anti-HER2 therapy with improved prognosis [13]. The majority of the basal-like subtype have a triple-negative phenotype. However, molecular profiling of breast cancer is expensive and not routinely available in breast pathology, and instead, immunohistochemical assessment of ER, PR, HER2 and Ki67 is used for surrogate subtype classification of the intrinsic molecular subtypes [5, 9, 14, 15]. Among HR+/HER2− tumors, Ki67 is important to distinguish luminal A-like and luminal B-like tumors and thereby the need for added chemotherapy [16, 17].

Intra- and interlaboratory variability of Ki67 assessment is known to hinder its reproducibility [18, 19]. International recommendations for Ki67 are controversial, due to lack of standardization, and as a consequence, laboratory-specific cut-off values have been recommended [5]. Despite efforts over the past years to establish robust recommendations, there is no international consensus regarding Ki67 cut-offs and the most appropriate method for Ki67 scoring [15, 20, 21]. International guidelines state that 1000 tumor cells should be counted, with an absolute minimum of 500 cells [5, 20]. In contrast, the national Swedish guidelines have concluded that 200 tumor cells should be counted in a hot spot region [22].

Digital image analysis (DIA) has been suggested as a method to improve reproducibility of Ki67, which has been demonstrated in several studies [23–25]. It was previously shown that DIA of Ki67 outperforms manual assessment and specifically the ability of DIA of Ki67 in hot spots to distinguish between luminal A- and B-like disease [26, 27]. The International Ki67 in Breast Cancer Working Group (IKWG) suggests automated average Ki67 scoring methods based on reproducibility, but states that the methods require further standardization and clinical validation [24].

A precise definition of a hot spot for Ki67 scoring is lacking in international guidelines, as well as recommendation for which assessment method to use [5, 15, 28]. The aim of this study was to compare the prognostic potential for Ki67 hot spot scoring and global scoring using different DIA platforms among ER+/HER2− breast cancers.

## Materials and methods

### Breast cancer study cohort

This retrospective study comprised a previously published cohort of patients diagnosed with invasive breast carcinoma at the Karolinska University Hospital, Sweden during 2002–2009 and the Stockholm South General Hospital, Sweden during 2012 [26, 27, 29, 30]. From this cohort, a total of 217 tumors were available for DIA (Supplementary Fig. S1). Clinicopathological data including up to 15 years of follow-up outcome data was retrieved from the pathology laboratory information system and the medical record system. Recurrence-free survival (RFS) was defined as no breast cancer recurrence at end of follow-up. Overall survival (OS) was defined as no death from any cause at end of follow-up. The “Reporting recommendations for tumor marker prognostic studies (REMARK)” were followed [31].

### Immunohistochemistry

Tissue serial sections were retrieved from formalin-fixed paraffin-embedded tumors at the accredited clinical laboratory of the Department of Pathology, Karolinska University Laboratory, Sweden. The sections were serially stained with a rabbit monoclonal anti-Ki67 antibody (clone 30-9) by Ventana and a mouse monoclonal anti-CKMNF116 antibody by Agilent Dako, according to manufacturer’s protocol, as described previously [27].

### Ki67 cut-offs and surrogate subtype classification

For assessment of Ki67 scoring methods and prognostic potential only ER+/HER2− luminal A-like and B-like tumors were included in the analysis. We adopted the St Gallen 2013 consensus recommendations for immunohistochemistry (IHC)-based surrogate subtype classification with a < 20% cut-off for low Ki67 [5]. Luminal A-like was defined as ER+/HER2− with PR ≥ 20% and low Ki67. Consequently, luminal B-like (non-HER2) was defined as ER+/HER2− with PR < 20% or high Ki67, as previously described by Robertson et al*.* [32]. HER2+ tumors were excluded since therapy choices for this tumor group is not primarily determined by proliferation index.

### PAM50 gene expression-based subtypes

For comparisons with molecular intrinsic subtypes available data on PAM50 gene expression-based subtypes were used. RNA extraction for gene expression analysis had been performed on snap-frozen tumor tissue as described previously [29, 30]. Based on the PAM50 algorithm, tumors had been assigned a molecular subtype (luminal A, luminal B, HER2-enriched or basal-like). No new gene expression analysis was performed for this study.

### Digital image analysis platforms

Digitalized whole slide images of tumor sections of Ki67 and CKMNF116 had previously been scanned with the NanoZoomer 2.0-HT (Hamamatsu Photonics K.K., Hamamatsu, Japan) platform at 20x, with a pixel size of 0.4537 × 0.4537 µm. Automated DIA algorithms for hot spot scoring were designed in the Visiopharm Integrator Software (VIS) (Visiopharm A/S, Hoersholm, Denmark). For global Ki67 scoring the open source software QuPath was used [33].

### Ki67 hot spot analysis

**Fig. 1:**
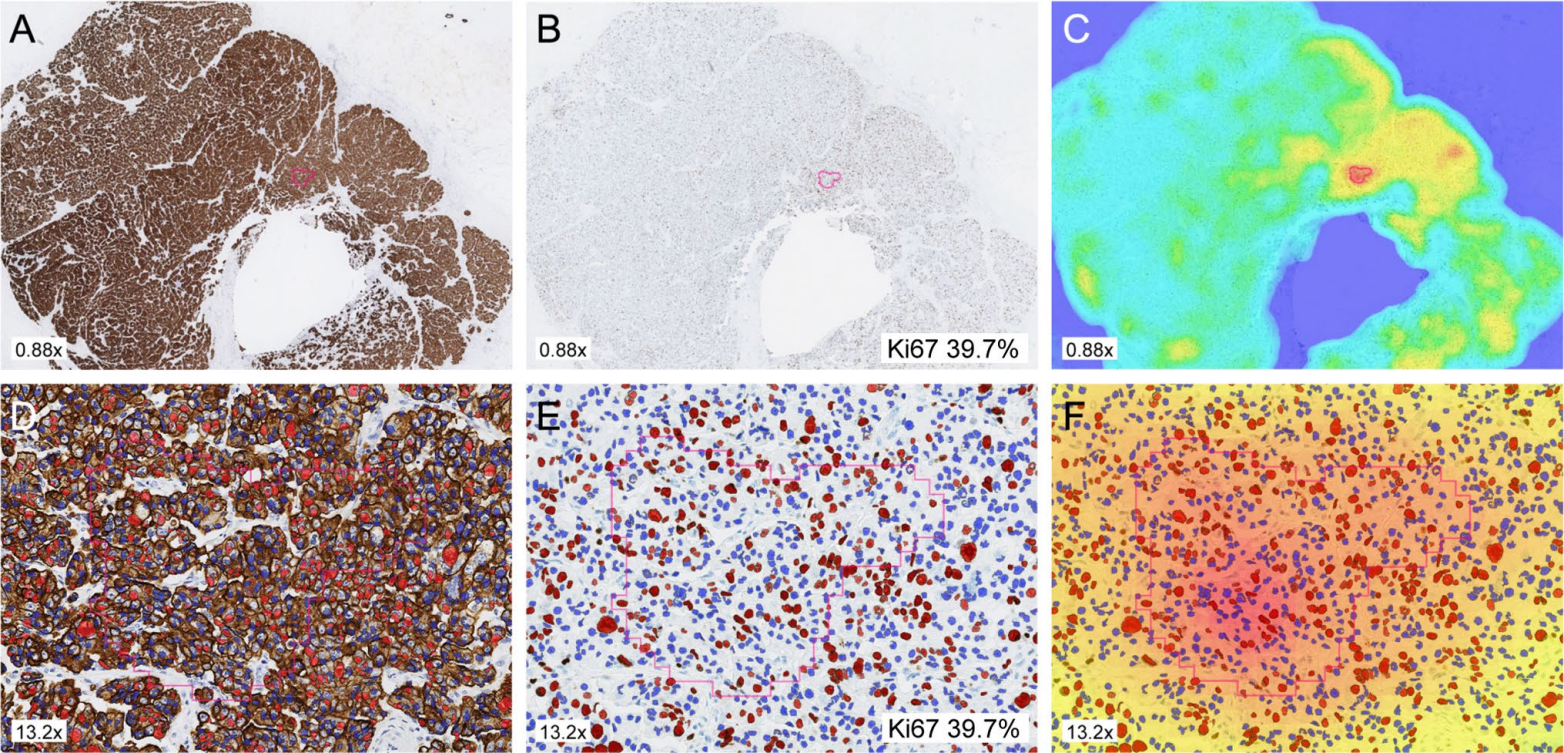
CKMNF116 (**a**) and aligned Ki67 (**b**) immunohistochemistry stained tumor slide with automated hot spot detection (pink outline) using APP24. Corresponding heatmap illustrating the areas with highest Ki67% score (**c**). Tumor cell detection and hot spot region visualized in CKMNF116 (**d**), Ki67 (**e**) and heatmap (**f**). Ki67-positive cells marked in red and Ki67-negative cells in blue

The Ki67-stained images were aligned with the CKMNF116-stained images in VIS using the Tissuealign module (Fig. 1). The tumor region detection operates by a VirtualDoubleStaining™ method, and accurately detects tumor cells (including non-invasive tumor components) and excludes non-epithelial cells e. g. proliferating lymphocytes and background tissue. Automated detection of tumor regions of interest (ROI) was performed using the pancytokeratin (PCK) VirtualDoubleStaining™ APP (ID: 10165) and Ki67 index (%) was estimated using the CE-IVD approved Ki67 APP (ID: 90004) identifying positive and negative tumor cell nuclei within the tumor regions. The PCK and Ki67 APP have previously been calibrated to the staining protocol and platform used at our department [27]. A hot spot was identified by applying the CE-IVD approved Hot Spot APP (ID: 20114, ver. 0.2) which is based on a heatmap of the density of Ki67-positive nuclei. Ki67 quantification (%) within the hot spot was performed by counting the number of positive nuclei divided by the total number of nuclei (Fig. 1). All images were reviewed by a pathologist and larger areas of non-invasive tumor within the ROIs were removed and all hot spots were confirmed to be in invasive ROIs.

### Hot spot parameters

We investigated different configurable parameters of the Hot Spot APP in VIS. The four identified parameters were the drawing radius, shape, positive cells or positive ratio, and total number of cells (Table 1). The hot spot was based on a heatmap using either the number of Ki67-positive cells or the ratio of positive cells in the tumor. The heatmap was generated by first creating an empty image at a much lower resolution than the virtual slide, with 0’s in all pixels. Then for each positive object in the image we added 1 to the heatmap image in a predefined drawing radius. The higher the radius, the more blurred heatmap, and the more round and cohesive the hot spot would be. We applied either a 20× or a 40× field of view, which generated a diameter of 1.04 mm or 0.52 mm, with a radius of 0.52 mm or 0.26 mm, respectively.

**Table 1:** Hot spot app characteristics with the four configurable parameters: number of cells in the hot spot, heatmap and drawing radius (20× or 40× field of view), heatmap based on positive cells or on positive ratio, and hot spot shape (circle or contour heatmap)

Hot spot app	Tumor cell count	Radius	Positive/ratio	Shape
APP02	200	40×	Pos	Circle
APP03	1000	20×	Pos	Circle
APP04	200	20×	Pos	Circle
APP05	1000	20×	Ratio	Circle
APP06	200	20×	Ratio	Circle
APP08	200	40×	Ratio	Circle
APP09	1000	20×	Pos	Contour heatmap
APP10	200	20×	Pos	Contour heatmap
APP11	1000	40×	Pos	Contour heatmap
APP12	200	40×	Pos	Contour heatmap
APP13	1000	20×	Ratio	Contour heatmap
APP14	200	20×	Ratio	Contour heatmap
APP15	1000	40×	Ratio	Contour heatmap
APP16	200	40×	Ratio	Contour heatmap
APP20	400	40×	Ratio	Contour heatmap
APP21	600	40×	Ratio	Contour heatmap
APP22	800	40×	Ratio	Contour heatmap
APP23	1200	40×	Ratio	Contour heatmap
APP24	400	40×	Pos	Contour heatmap
APP25	600	40×	Pos	Contour heatmap
APP26	800	40×	Pos	Contour heatmap
APP27	1200	40×	Pos	Contour heatmap

The ratio heatmap takes both Ki67-positive and negative tumor cells into account, and a threshold can be set to indicate the minimum number of cells needed for it to be considered a hot spot. This can then be combined with the heatmap to only show hot spots with the set minimum number of cells. Notably, ratio heatmaps can have tendencies to show hot spots at the periphery of the tissue: partly putting the hot spot on the background area for the criteria to be met.

The two most relevant methods to set up the shape of the hot spot was by creating a circular hot spot or a hot spot that follows the contours of the heatmap. The circular hot spot corresponds to the field of view through a microscope. The contour heatmap hot spot also allows the hot spot to more closely follow the heatmap, and a smaller drawing radius should in general then be used.

The number of cells in the hot spot is influenced by several parameters. The heatmap can be limited to only show hot spots in areas with a minimum number of cells. As the area of the hot spot is fixed, the number of cells will vary depending on the tumor density, but a minimum number can be guaranteed through heatmap limiting. According to current guidelines, we initially set the minimum number of cells to either 200 or 1000 cells.

These four defined parameters were combined into 16 hot spot apps, namely APP01-16. APP01 and APP07 were excluded before analysis since the combination of 1000 cells and 40× radius was not appropriate here. Furthermore, additional APP20-27 were created combining either 400, 600, 800 or 1200 cells, and a total of 22 hot spot apps were created (Table 1). Each hot spot app provided a Ki67 score from a single hot spot for every tumor case it was run on. Depending on the app parameters, the location of the hot spot could vary across the tumor area for different apps run on the same tumor. Thus, the hot spot location may be either central or peripheral.

### Ki67 global scoring

**Fig. 2:**
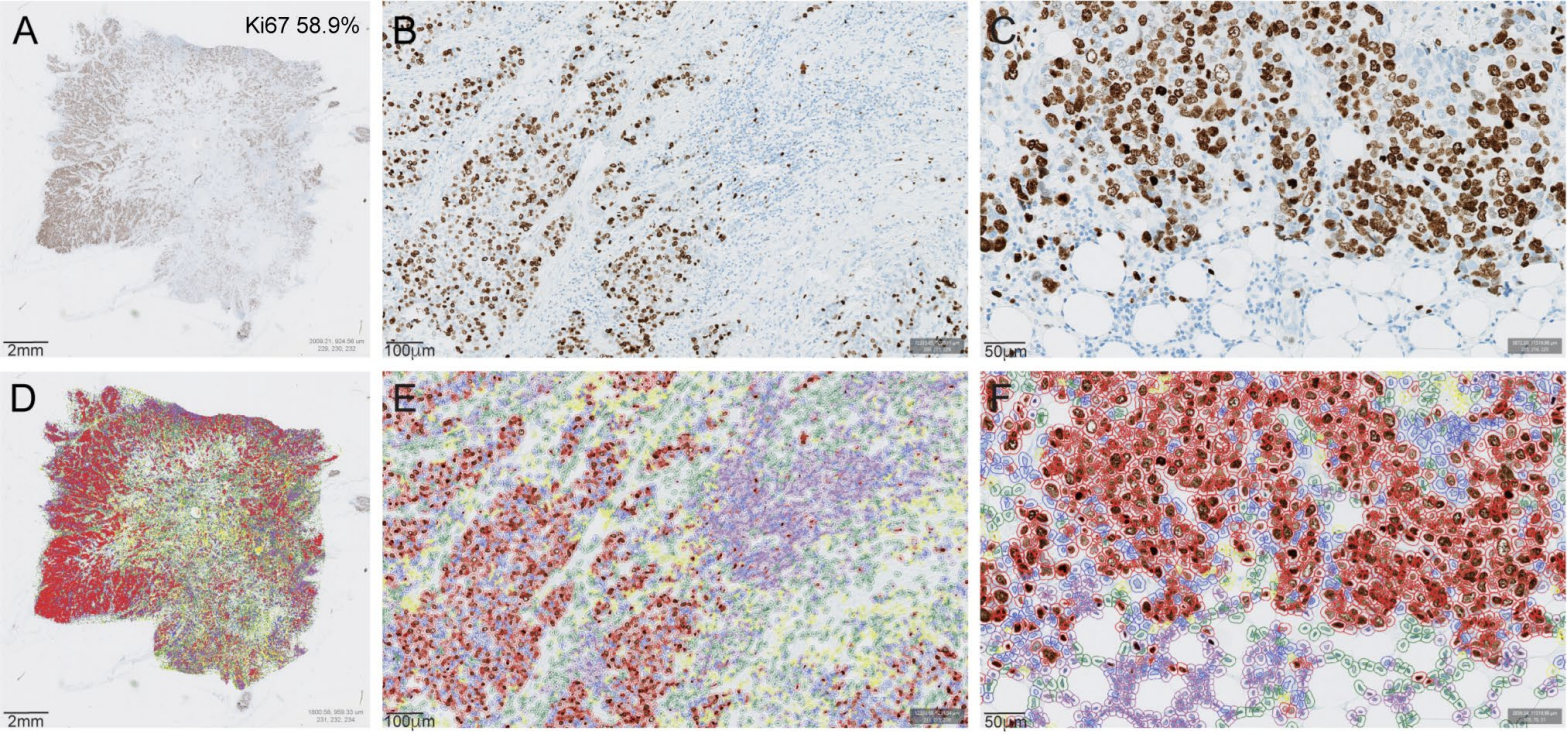
Automated Ki67 scoring algorithm in QuPath illustrated by a Ki67 immunohistochemistry stained tumor slide (**a**–**c**) with the cell classifier (**d**–**f**) for global Ki67 scoring. Ki67-positive tumor cells marked in red, Ki67-negative tumor cells in blue, immune cells in purple, stromal cells in green and other cells in yellow (**d**–**f**)

The QuPath (open source software [33]) platform was used to build an automated Ki67 scoring algorithm for the general Ki67 scoring in breast cancer. As the date of Ki67 staining varied within the cohort, we refined the immunohistochemical and hematoxylin stain estimates for each digitized slide (estimate stain vectors command in QuPath). We used watershed cell detection [34] to segment the cells in the image with the following settings: detection image, optical density sum; requested pixel size, 0.5 µm; background radius, 8 µm; median filter radius, 0 µm; sigma, 1.5 µm; minimum cell area, 10 µm^2^; maximum cell area, 400 µm^2^; threshold, 0.1; maximum background intensity, 2. In order to classify detected cells into tumor cells, immune cells, stromal cells and others (false detections, background), we used random trees as a machine learning method [35] (Fig. 2). The features used in the classification are described in Supplementary Table S1. In order for the algorithm to perform an accurate classification, we also added smoothed object features at 25 and 50 µm radius to supplement the existing measurements of individual cells. The quality control of the algorithm to classify detected cells was performed by a pathologist. The analysis was run on the entire tumor area on the whole slide defined by a pathologist and output as a global Ki67 score (%).

For global scoring the algorithm was trained only on Ki67 immunohistochemical staining and the training was performed on 500 cells in an independent training cohort of 30 ER+ breast cancer tumors. Regarding global Ki67 scoring, a ≥ 20% cut-off was used for distinguishing high from low proliferation as recommended by the St Gallen 2013 [5].

### Statistical analysis

Normal distribution was tested by Kolmogorov–Smirnov test of normality, and non-parametric methods were used for significance testing. The intraclass correlation coefficient was used to test reproducibility using log-transformed Ki67 values. The agreement between Ki67 values by DIA hot spot and DIA global scoring was assessed in a Bland–Altman plot. The Kaplan–Meier method was used for survival analysis of OS and RFS, and compared using log-rank test. The Cox proportional hazard model for univariate and multivariate analysis was used for analysis of prognostic potential. McNemar test for categorical paired variables and Cohen’s *κ* test for scoring and subtype agreement were used. The statistical analysis was performed using IBM SPSS Statistics version 25 (IBM Corporation, Armonk, NY, USA). *p* values < 0.05 were considered significant. Power analysis was calculated and was set to ≥ 0.80.

## Results

**Fig. 3:**
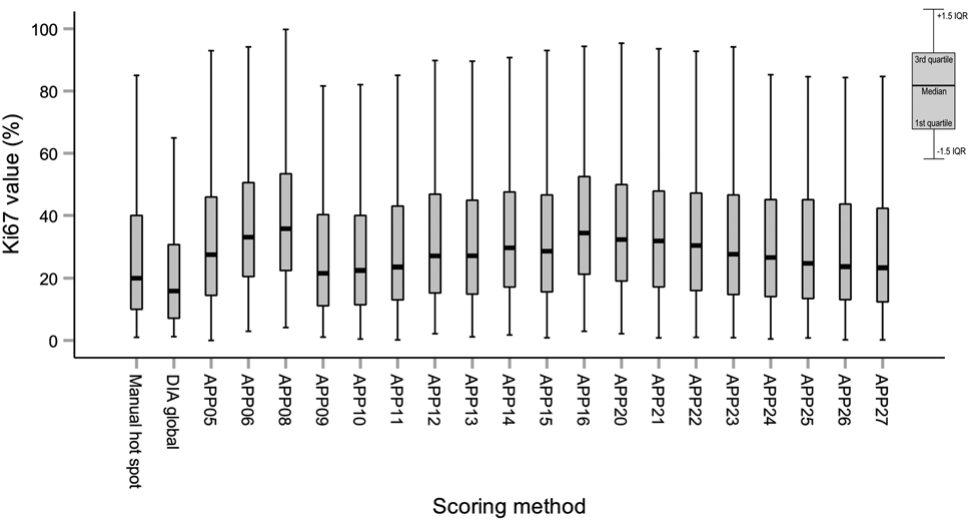
Ki67 distribution for luminal-like tumors (*n* = 139) scored by manual hot spot, DIA global or DIA hot spot apps (APP05-27). Box plot illustrating median, interquartile range and range. *DIA* digital image analysis, *IQR* interquartile range

Of the 217 tumors available for DIA, a total of 48 cases were excluded after strict criteria and pathologist review (Supplementary Fig. S1). The excluded cases were either due to no invasive tumor in slide (*n* = 2), poor immunohistochemical staining (*n* = 4), misalignment (*n* = 2), hot spot detected in artifacts (*n* = 16) or in ductal carcinoma in situ components (*n* = 11), or other errors in analysis (*n* = 13). Only cases with successful DIA scores for all 22 apps were included for further analysis (*n* = 169). Among these cases, 139 were identified as ER+/HER2-, thus classified as luminal A-like or luminal B-like (HER2-) tumors and included in all further analysis (Table 2). The median follow-up time for RFS was 8.7 years (range 0.3–14.7 years) and 9.1 years for OS (range 2.1–14.8 years). The median Ki67 score by DIA hot spot apps ranged from 21.6 to 35.7%. The median Ki67 score by manual and DIA global scoring was 20.0% and 15.9%, respectively (Fig. 3).

**Table 2:** Patient and tumor characteristics

	No.	%
Total no. of tumors	139	100
Patient mean age at diagnosis (years)	59	–
Histological subtype		
Ductal/no special type	112	80.6
Lobular	16	11.5
Other	11	7.9
Nottingham histological grade		
Grade 1	21	15.1
Grade 2	72	51.8
Grade 3	44	31.7
Unclassified	2	1.4
Tumor size (mm) and pT*		
≤ 20, pT1	55	39.6
> 20 and ≤ 50, pT2	76	54.7
> 50, pT3	8	5.8
No. of positive lymph nodes and pN*		
0, pN0	76	54.7
1–3, pN1	45	32.4
4–9, pN2	13	9.4
≥ 10, pN3	5	3.6
Estrogen receptor (%)		
≥ 1 and < 10	0	0.0
≥ 10	132	95.0
Positive by other method	7	5.0
Progesterone receptor (%)		
< 20	36	25.9
≥ 20	99	71.2
Positive by other method	4	2.9
Ki67 (%)		
< 20	59	42.4
≥ 20	70	50.4
Unknown numerical value	10	7.2
IHC-based surrogate subtype		
Luminal A-like	47	33.8
Luminal B-like (HER2−)	82	59.0
LumA/LumB unknown Ki67 value	10	7.2
PAM50 intrinsic subtype		
Luminal A	79	56.8
Luminal B	30	21.6
HER2-enriched	1	0.7
Basal-like	1	0.7
Unclassified	28	20.1
Neoadjuvant treatment		
Endocrine therapy	1	0.72
Adjuvant treatment		
Chemotherapy	54	38.8
Endocrine therapy	118	84.9
Radiotherapy	89	64.0
Anti-HER2 therapy	0	0.0
Unclassified	18	12.9
Outcome		
Patients with recurrence at end follow-up	28	20.1
5-year recurrence-free survival rate (%)		82.9
10-year recurrence-free survival rate (%)		57.1
Patients dead at end follow-up	20	14.4
5-year overall survival rate (%)		94.3
10-year overall survival rate (%)		67.7

*Pathologic T stage (pT) for invasive tumor and pathologic N stage (pN) for regional lymph nodes according to AJCC Breast Cancer Staging 7th Edition (TNM 7)

### Automated Ki67 scoring

**Fig. 4:**
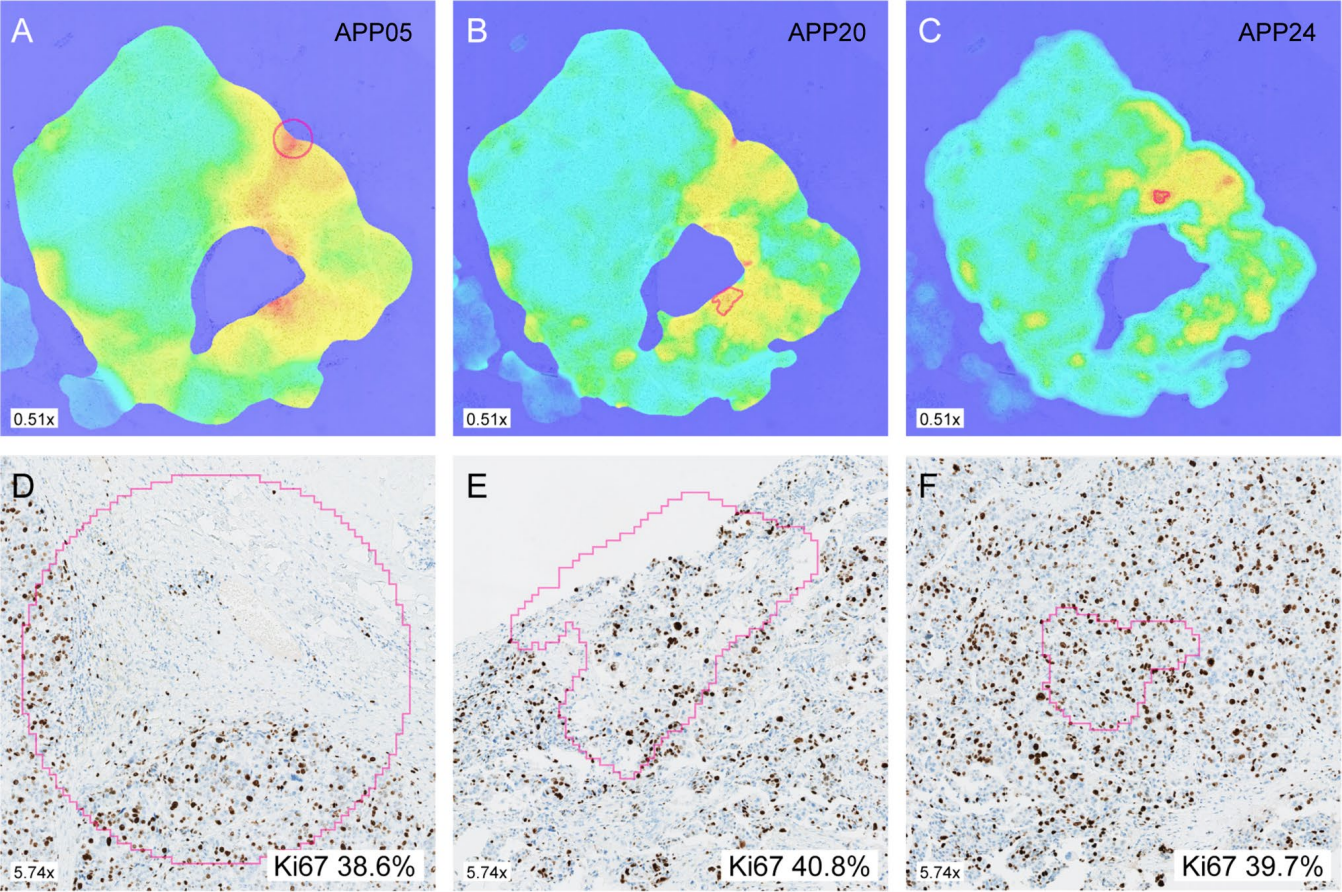
Heatmaps from the same tumor showing different Ki67 hot spot areas (pink outline) using APP05 (**a**), APP20 (**b**) and APP24 (**c**). Hot spots (pink outline) with Ki67 values for each corresponding app (**a**–**c**) are illustrated in **d**–**f**, respectively

**Fig. 5:**
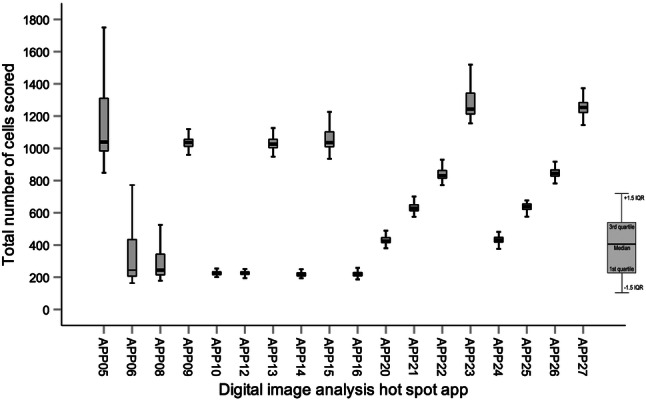
Box plot illustrating the distribution of the number of cells scored for each hot spot app included. *IQR* interquartile range

Applying different hot spot apps on the same tumor whole slide image shows variations in heatmap pattern and region of detected hot spot as illustrated in Fig. 4. The distribution of number of cells scored for each app included is shown in Fig. 5 and Supplementary Fig. S2. The extreme outliers APP03 (median 2366 cells, range 76–5965 cells) and APP04 (median 2366 cells, range 588–5965 cells) were excluded since the range of number of cells far exceeded the set included cell count of 1000 and 200 cells, respectively. APP02 was also excluded due to cell count far exceeding the defined 200 cells (median 715 cells, range 199–1629 cells). After exclusion, 19 different apps remained for analysis.

The intraclass correlation coefficient among apps with low cell counts around 200 cells (APP06, 08, 10, 12, 14, 16) was 0.858 [confidence interval (CI) 0.768–0.918]. For apps with 400–800 cells (APP20, 21, 22, 24, 25, 26), the intraclass correlation coefficient was 0.956 (CI 0.919–0.973), and 0.959 (CI 0.935–0.973) among apps with high cell counts, around 1000 cells (APP05, 09, 13, 23, 27). Consequently, the higher the scored cell counts, the greater the reproducibility of Ki67 values. The median tumor cell counts with DIA global scoring in QuPath was 97,940 cells (range 9550–1,055,427 cells). A Bland–Altman plot for comparison of the agreement of Ki67 values by hot spot APP24 and DIA global scoring showed systematic differences between the two methods (*p* < 0.0001; regression slope *p* < 0.0001, intercept *p* = 0.0004; Supplementary Fig. S3).

### Prognostic potential for hot spot versus global scoring

**Fig. 6:**
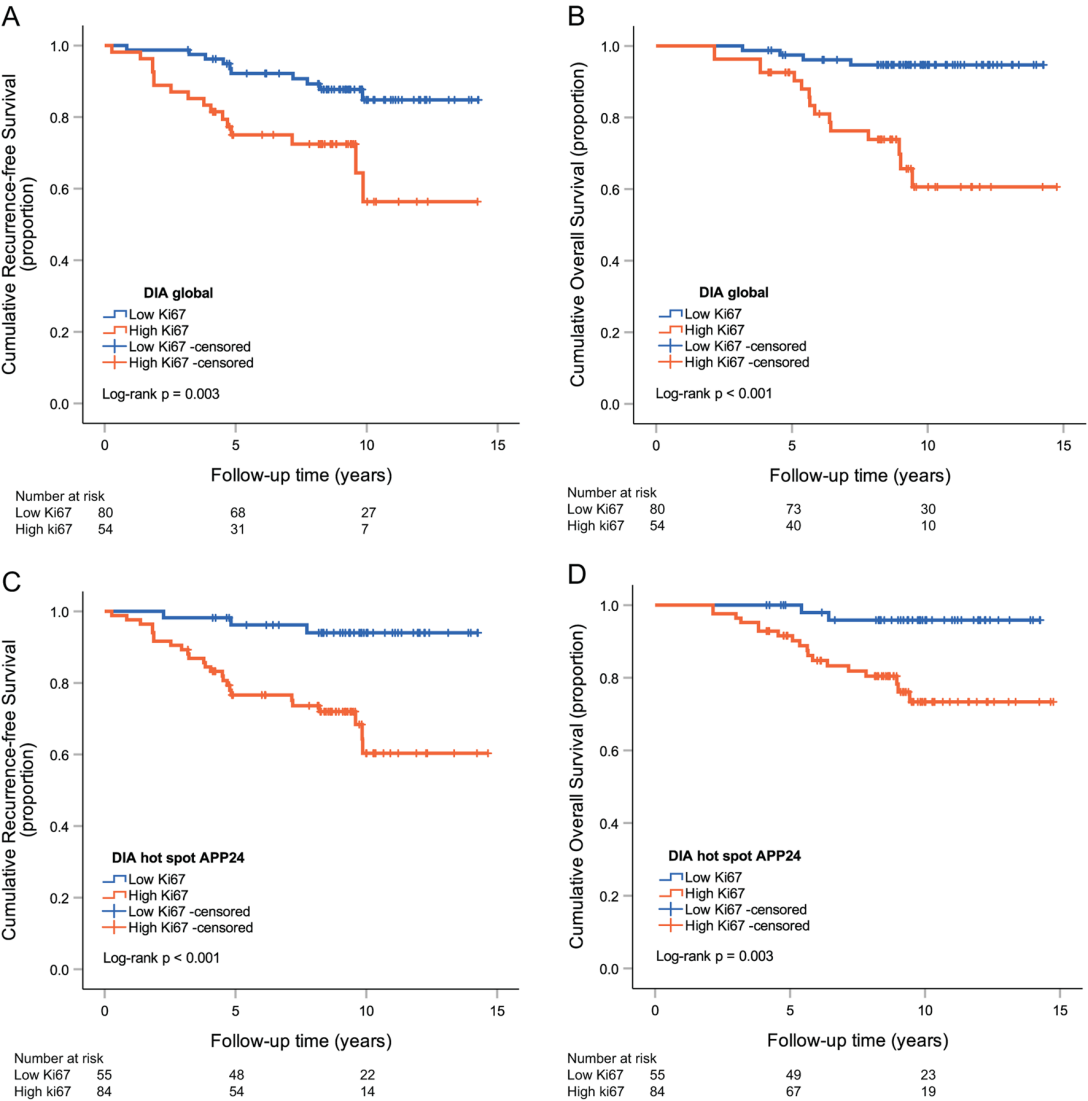
Kaplan–Meier curves demonstrating associations between low versus high Ki67 subgroups and recurrence-free survival (**a**) or overall survival (**b**) using global scoring. Kaplan–Meier curves demonstrating associations between low versus high Ki67 subgroups and recurrence-free survival (**c**) or overall survival (**d**) using hot spot APP24 Ki67 scoring. *DIA* digital image analysis

Regarding prognostic potential, the following apps showed the highest hazard ratios (HR) for RFS: APP10, 11, 15, 23, 24 and 26 (Supplementary Table S2). APP24 reached the highest prognostic potential among hot spot apps for RFS (HR 6.88, CI 2.07–22.87, *p* = 0.002), compared to global Ki67 scoring with a HR of 3.13 (CI 1.41–6.96, *p* = 0.005) (Fig. 6). Cox regression HR for OS and high versus low Ki67 was highest with APP27 (HR 8.42, CI 1.95–36.35, *p* = 0.004); however, APP24 (HR 6.93, CI 1.61–29.91, *p* = 0.009) was slightly inferior to global Ki67 scoring (HR 7.46, CI 2.46–22.58, *p* < 0.001) (Supplementary Table S2). Manual Ki67 hot spot scoring was only significant for RFS (HR 2.76, CI 1.16–6.53, *p* = 0.021) (Supplementary Table S2 and Fig. S4).

The prognostic value was further investigated among node-negative (pN0) patients and those with 1–3 axillary lymph node metastases (pN1). Survival analysis with Kaplan–Meier estimates showed significant difference in OS and RFS among pN0 cases with high versus low Ki67 scored by the global scoring method and in RFS using hot spot APP24 (Supplementary Fig. S5, S6). Further, among pN0 cases, the HR for RFS was significantly increased in high versus low Ki67 cases scored by the global method (HR 4.12, CI 1.01–16.74, *p* = 0.048). No significant differences in HR for RFS among pN1 cases or in OS among pN0 and pN1 patients was shown by any scoring methods (Supplementary Table S3 and Fig. S5, S6). When cases were stratified for grade 1 tumors, no increased HR for OS (APP24 HR 0.04, *p* = 0.756; global HR 0.04, *p* = 0.814) was identified and notably all grade 1 cases were free from recurrence. We also stratified for mitotic score 1 (*n* = 62), and the HR for RFS was significantly increased in high versus low Ki67 cases using DIA hot spot scoring (HR 5.05, CI 1.26–20.25, *p* = 0.022), but not with DIA global scoring (HR 5.01, CI 0.90–27.95, *p* = 0.066). Here, there was no significant increased risk for death in Ki67 high vs low cases using any of the scoring methods (APP24 HR 6.80, *p* = 0.97; global HR 3.94, *p* = 0.263).

Kaplan–Meier analysis for RFS with hot spot Ki67 scoring reached a power of 0.90 and a power of 0.95 for OS with global scoring. Global scoring for RFS (power < 0.80) and hot spot scoring for OS (power < 0.80) was not considered powered enough.

### IHC-based surrogate subtypes versus PAM50 subtypes

PAM50 intrinsic subtypes were available for 111 tumors out of which 79 were luminal A (71.2%), 30 luminal B (27.0%), one was basal-like (1.0%) and one was HER2-enriched (1.0%; Table 2). For subtype comparisons, only luminal A and B tumors were included (*n* = 109). We used hot spot APP24 Ki67 scores and global Ki67 scores for IHC-based surrogate subtype classification with a 20% cut-off for Ki67. Based on Ki67 values from hot spot APP24, 39 tumors were classified as luminal A-like (30.0%) and 93 tumors as luminal B-like (70.5%). Among 104 tumors with PAM50 subtype data and surrogate subtype based on hot spot Ki67 scores, 58 tumors had concordant subtype (55.8%, *κ*  = 0.250). When global Ki67 values were used, 62 tumors were classified as luminal A-like (48.4%) and 66 as luminal B-like (51.6%). Here, among 101 tumors with PAM50 subtype and surrogate subtype based on global Ki67 scores, 67 tumors hade concordant subtype (66.3%, *κ*  = 0.345).

Patients with luminal B tumors (PAM50 subtype) had significantly higher hazard ratios for recurrence (HR 2.636, CI 1.180–5.892, *p* = 0.018) and death (HR 4.050, CI 1–541–10.647, *p* = 0.005) as compared to those with luminal A tumors (Supplementary Fig. S7). When tumors were divided in luminal A-like and luminal B-like using hot spot Ki67, Kaplan–Meier estimates showed a significant worse RFS (log-rank *p* = 0.002) and OS (log-rank *p* = 0.011) for patients with luminal B-like tumors (Supplementary Fig. S7). The HR was 12.351 for RFS (CI 1.676–91.032, *p* = 0.014) and 8.648 (CI 1.158–64.6, *p* = 0.035) for OS in luminal B-like versus luminal A-like cases, and no further conclusions are made due to the broad CI. Applying global Ki67 for surrogate subtypes, did not provide any significant difference between luminal B-like and luminal A-like cases with regard to RFS (HR 1.899, CI 0.834–4.302, *p* = 0.124). On the other hand, there was a significantly increased HR for OS (HR 5.947, CI 1.731–20.434, *p* = 0.005) in luminal B-like versus luminal A-like cases (Supplementary Fig. S7).

### Multivariate Cox regression analysis

To further investigate the individual prognostic potential of hot spot APP24, DIA global and manual hot spot scoring, we performed a multivariate Cox regression analysis. The categorical covariates tumor size (pT1, pT2, pT3), tumor Nottingham histological grade (1, 2, 3), mitotic score (1, 2, 3) and lymph node status (pN0, pN1 or pN0, pN1, pN2, pN3, respectively) were tested in univariate Cox regression analyses, out of which only lymph node status including pN0/1/2/3 was significantly (*p* = 0.005) associated to RFS (Supplementary Table S4). Regarding the clinically relevant pN0 and pN1 cases, lymph node status was, however, not significant in univariate analysis (*p* = 0.208). A multivariate Cox proportional hazards regression model was fitted to RFS time of the 139 cases. Adjusting the model to lymph node status (pN0/1), DIA global scoring (HR 3.53, CI 1.21–9.54, *p* = 0.013) and manual hot spot scoring (*p* = 0.036) remained significantly associated with RFS (Table 3). In the multivariate model, the HR for RFS using DIA hot spot scoring resulted in an unreliably broad CI (HR 13.80, CI 1.83–104.05, *p* = 0.011). Adding lymph node status including pN2/3 cases to the multivariate model, all scoring methods remained significantly associated with RFS (APP24 *p* = 0.001, global DIA *p* = 0.004 and manual hot spot *p* = 0.022) (Supplementary Table S5).

**Table 3:** Multivariate Cox proportional hazard models for recurrence-free survival

Variables in model		Hazard ratio	95% confidence interval	*p*
pN stage^a^ (pN1 vs pN0)		1.99	0.79–5.03	0.144
Ki67 DIA hot spot APP24 scoring (≥ 20% vs < 20%)		13.80	1.83–104.05	0.011*
pN stage (pN0 vs pN1)		1.54	0.61–3.90	0.361
Ki67 DIA global scoring (≥ 20% vs < 20%)		3.53	1.31–9.54	0.013*
pN stage (pN0 vs pN1)		1.44	0.57–3.65	0.438
Ki67 manual hot spot scoring (≥ 20% vs < 20%)		3.31	1.08–10.11	0.036*

*DIA* digital image analysis

*p significant at a < 0.05 level

^a^Pathologic N stage for regional lymph nodes according to AJCC Breast Cancer Staging 7th Edition (TNM 7)

Turning to OS, only the categorical covariates tumor grade (1, 2, 3) and mitotic score (1, 2, 3) was significantly associated with OS in univariate analysis (*p* < 0.001 and *p* = 0.009, respectively). Regarding the clinically relevant pN0 and pN1 cases, lymph node status was not significant in univariate analysis (*p* = 0.114; Supplementary Table S6). The multivariate Cox regression model was fitted to OS time, adjusting for grade, mitotic score and lymph node status (pN0/1). When each of the three Ki67 scoring methods was added to the model, only global Ki67 scoring remained significant (HR 7.11, CI 1.09–46.46, *p* = 0.040) in the multivariate analysis associated to OS (Table 4). The HR for OS using DIA global scoring remained significant and with a narrower CI in the model adjusted for only grade and mitotic score (HR 5.44, CI 1.15–25.69, *p* = 0.032).

**Table 4:** Multivariate Cox proportional hazard models for overall survival

Variables in model	Hazard ratio	95% confidence interval	*p*
Grade (1 ref)	–	–	–
Grade 2	0.92	0.08–10.80	0.949
Grade 3	10.55	0.39–287.98	0.163
Mitotic score (1 ref)	–	–	–
Mitotic score 2	0.42	0.04–4.85	0.487
Mitotic score 3	0.21	0.01–3.64	0.284
pN stage^a^ (pN1 vs pN0)	2.28	0.73–7.14	0.158
Ki67 DIA hot spot APP24 scoring (≥ 20% vs < 20%)	2.93	0.50–17.35	0.236
Grade (1 ref)	–	–	–
Grade 2	1.05	0.09–11.62	0.971
Grade 3	6.25	0.23–171.59	0.278
Mitotic score (1 ref)	–	–	–
Mitotic score 2	0.30	0.03–3.70	0.348
Mitotic score 3	0.19	0.01–3.40	0.261
pN stage^a^ (pN1 vs pN0)	1.87	0.61–5.75	0.277
Ki67 DIA global scoring (≥ 20% vs < 20%)	7.11	1.09–46.46	0.040*
Grade (1 ref)	–	–	–
Grade 2	1.09	0.10–12.06	0.944
Grade 3	13.32	0.51–348.73	0.120
Mitotic score (1 ref)	–	–	–
Mitotic score 2	0.70	0.06–8.23	0.778
Mitotic score 3	0.50	0.03–9.40	0.645
pN stage^a^ (pN1 vs pN0)	1.60	0.51–5.01	0.423
Ki67 manual hot spot scoring (≥ 20% vs < 20%)	0.72	0.20–2.65	0.620

*DIA* digital image analysis

*p significant at a < 0.05 level

^a^Pathologic N stage for regional lymph nodes according to AJCC Breast Cancer Staging 7th Edition (TNM 7)

### Categorical Ki67 score comparison

McNemar test for categorical paired variables showed significant difference between DIA hot spot APP24 and global Ki67 scorings (*p* < 0.001). The agreement for low and high Ki67 grouping using hot spot and global scoring showed a *κ* value of 0.54, referred to as moderate agreement.

## Discussion

We compare several different DIA hot spot apps with DIA global scoring using virtual dual staining versus traditional immunohistochemistry for DIA in a cohort of luminal-like tumors. Despite the established prognostic and predictive value of Ki67 for patients with HR+/HER2− tumors [4, 36], there is a lack of international expert consensus regarding assessment methods and standardization for Ki67 evaluation [5, 17, 20]. Pre-analytical and analytical aspects along with poor interlaboratory scoring reproducibility are some of the identified causes of variability in Ki67 assessment, which has limited the international adoption in clinical breast cancer management [18, 19, 21]. There is increasing evidence suggesting that global or average scoring of Ki67 is favorable over hot spot scoring methods, and here Leung et al*.* suggest against the use of manual Ki67 hot spot scoring due to poor reproducibility [37, 38]. The IKWG also point to the methodological aspects for improvement of Ki67 assessment [24, 38]. In a study by Jang et al*.* manual average and hot spot methods for Ki67 scoring among HR+/HER2− tumors was compared and both methods showed good predictive performances for recurrence; however, the average method showed higher reproducibility [39].

The European Society of Medical Oncology Clinical Practice Guidelines point out the importance of standardization of Ki67 scoring. By recommending IHC-based surrogate intrinsic subtype classification of tumors they indirectly imply the use of Ki67 [40]. The St Gallen consensus of 2019 supports the use of gene expression signature assays for patients with ER+ tumors with < 3 positive lymph nodes to determine the benefit of additional chemotherapy. When gene expression signatures are not available, surrogate subtyping may be based on a combination of grade, ER/PR and Ki67 [41]. The recommendations from the Breast Committee of the German Gynecological Oncology Group (AGO) are in line, and do not provide any specific guidelines for Ki67 scoring but mention Ki67 for distinguishing luminal B-like tumors [42]. On the contrary, the American Society of Clinical Oncology Clinical Practice Guideline concludes that there is insufficient evidence to recommend the use of Ki67 for choice of adjuvant chemotherapy [43, 44]. As proposed by the St Gallen consensus in 2013, laboratory-specific cut-offs for Ki67 are used in Sweden since 2018 [5]. According to Maisonneuve et al*.* Ki67 is categorized into three groups: low, intermediate and high proliferation, where PR has importance for dichotomizing the intermediate Ki67 group into luminal A-like and luminal B-like tumors [16]. This has been adopted by the Swedish guidelines and low Ki67 is defined as < 15%, intermediate Ki67 as 15–22% and high Ki67 ≥ 23% at our institution (Department of Clinical Pathology, Karolinska University Laboratory) [22]. Similar to the Swedish guidelines, the Danish Breast Cancer Cooperative Group recommends Ki67 to be scored in hot spots, but also in the invasive tumor fronts and in 5–10% intervals [45]. External quality assurance programs (e.g., NordiQC) for immunohistochemical assessments and frequent monitoring are important measures to continuously improve the quality of Ki67 scoring [46]

Computerized image analysis is rapidly emerging and has potential to improve biomarker assessment. We have previously reported that automated DIA for Ki67 scoring outperforms manual scoring, and that DIA hot spot Ki67 scoring was the superior method for distinguishing luminal A-like from B-like tumors [26, 27]. Apart from conventional machine learning methods, Saha et al*.* reported high precision using a deep learning approach for automated Ki67 hot spot scoring on immunohistochemically stained breast tumor images compared to manual scoring [47]. The reproducibility of automated scoring was recently investigated in a multicenter study by the IKWG and suggests that automated average Ki67 scoring methods hold promise but require standardization and clinical validation [24]. Furthermore, excellent reproducibility of Ki67 evaluation across different DIA platforms, including QuPath, has recently been shown, as well as how DIA can be standardized to improve Ki67 scoring [23].

In our study, we investigated different configurable parameters for defining a digital hot spot region with regards to prognostic potential. To date there is no clinically validated recommendations for hot spot definitions with automated scoring methods. When our DIA hot spot apps were grouped based on total cell counts, we show that the reproducibility of Ki67 scores depends on the investigated cell numbers. The larger the number of investigated cells, the higher the reproducibility between the apps in the group. The median Ki67 value was higher across all DIA hot spot apps (21–35%) and manual hot spot scoring (20%) as compared to the global DIA Ki67 scoring (15.9%), which is in line with previous published data [38].

The prognostic value of Ki67 can be used to distinguish patients in low and high Ki67 groups based on outcome. Among all the tested digital hot spot apps, our results showed that the selected DIA hot spot APP24, which was based on 400 cells, 40× field of view and a heatmap shaped hot spot, had twice as high hazard ratio for RFS compared to DIA global Ki67 scoring in univariate analysis. In this app (APP24), the hot spot was based on positive nuclei, which, however, does not consider the cell density. A dense area might contain a larger number of nuclei, and hence a larger number of positive nuclei, and have a lower percentage of positive cells than another more sparsely populated region. The heatmap shaped hot spot requires a minimum number of cells to be included. As the hot spot follows the shape of the heatmap it will sometimes include slightly more nuclei than the minimum number, but never less. Regarding HR for OS in the univariate model, DIA global scoring was superior to hot spot scoring, which was also shown among node-negative cases. Furthermore, adjusted multivariate models showed that DIA global scoring had independent prognostic value for both RFS and OS, which was not shown for DIA hot spot Ki67 scoring.

Molecular subtyping of tumors based on, e.g., the PAM50 algorithm provides prognostic information, which was also confirmed in this study. In our cohort, the concordance of DIA Ki67-based subtypes and PAM50 subtypes was rather low, thus slightly greater using global Ki67 values as opposed to hot spot scores. Using Ki67 values from both hot spot and global DIA scoring for IHC-based surrogate subtyping in luminal A-like and luminal B-like tumors, only the global Ki67 method provided prognostic value for OS.

There are certain limitations to the study. The study cohort size is limited, which affected the power especially regarding outcome analysis. With the strict inclusion criteria, even cases which failed in only one app were excluded from analysis. Despite different platforms and methods for Ki67 scoring, we applied the same cut-off of ≥ 20% to define high proliferation in both the hot spot and global scoring. Some known prognostic clinicopathological factors, such as lymph node status was not significant in multivariate analysis for OS, most likely due to the rather low number of cases in each category. Since lymph node status is one of the most powerful prognostic factors in breast cancer, it was valuable to add pN0/1 to the multivariate adjusted model for both RFS and OS. Moreover, the patient cohort consists of a combination of both pre- and postmenopausal patients (age ranged from 28 to 79 years), since this was not a predefined inclusion criterion. Prognostic information based on surrogate IHC markers are mainly relevant for postmenopausal patients, which may be spared chemotherapy for those with luminal A-like tumors [48–50]. Regarding clinical utility in routine pathology, virtual dual staining with parallel sections stained for Ki67 and pancytokeratins is impractical and does not add any further value to the diagnostic process. By using more specific cytokeratins, e.g., dual staining with CK5 and CK18 instead of CKMNF116, thus also providing information regarding in situ components, which is often part of the routine work-up, the use of virtual dual staining could potentially be feasible for Ki67 scoring.

Despite these limitations, to our knowledge, this is the first study investigating the effect of different hot spot definitions on both reproducibility and prognostic potential, along with comparing the prognostic value of true global scoring, using two separate DIA platforms. This study showed similar prognostic potential using DIA hot spot and global Ki67 scoring, but only DIA global scoring was independently significant in adjusted multivariate analysis for both RFS and OS. Overall, we showed robust outcome prediction with DIA global Ki67 scoring in this ER+/HER2− cohort. Regarding clinical routine, DIA global Ki67 scoring based on only Ki67-stained sections is a more practical method than the virtual dual staining method for hot spot scoring. Based on our findings we can conclude that automated global Ki67 scoring is feasible and shows clinical validity. However, these findings need to be confirmed in a larger study cohort to prove clinical utility leading to clinical implementation.

## Electronic supplementary material

Below is the link to the electronic supplementary material.

Supplementary file1 (PDF 1971 kb)
