# Supplementary material for: Prognostic potential of automated Ki67 evaluation in breast cancer: different hot spot definitions versus true global score
Source: Breast Cancer Res Treat. 2020 Jun 22;183(1):161–75. doi: 10.1007/s10549-020-05752-w (PMC7376512; doi:10.1007/s10549-020-05752-w)
Supplement: Supplementary file 1 — Supplementary file1 (PDF 1971 kb) [file 10549_2020_5752_MOESM1_ESM.pdf]

**ELECTRONIC SUPPLEMENRARY MATERIAL**

To

# Prognostic potential of automated Ki67 evaluation in breast cancer: different hot spot definitions versus true global score

Stephanie Robertson[1,2], Balazs Acs[1,2], Michael Lippert[3] and Johan Hartman[1,2]

[1]Department of Oncology and Pathology, Karolinska Institutet, Stockholm, Sweden.
[2]Department of Clinical Pathology and Cytology, Karolinska University Laboratory, Stockholm, Sweden.
[3]Visiopharm A/S, Hoersholm, Denmark.

Corresponding author: Stephanie Robertson, Department of Oncology and Pathology, Karolinska Institutet, CCK R8:04, 17176 Stockholm, Sweden.
e-mail: stephanie.robertson@ki.se

Journal: Breast Cancer Research and Treatment

**Content:**

**Table S1-S6**

**Fig. S1-S7**

**Table S1** List of features used in the random trees' classifier in QuPath

| | | |
|---|---|---|
| Nucleus: Area | Smoothed: 25 µm: Nucleus: Area | Smoothed: 50 µm: Nucleus: Area |
| Nucleus: Perimeter | Smoothed: 25 µm: Nucleus: Perimeter | Smoothed: 50 µm: Nucleus: Perimeter |
| Nucleus: Circularity | Smoothed: 25 µm: Nucleus: Circularity | Smoothed: 50 µm: Nucleus: Circularity |
| Nucleus: Max caliper | Smoothed: 25 µm: Nucleus: Max caliper | Smoothed: 50 µm: Nucleus: Max caliper |
| Nucleus: Min caliper | Smoothed: 25 µm: Nucleus: Min caliper | Smoothed: 50 µm: Nucleus: Min caliper |
| Nucleus: Eccentricity | Smoothed: 25 µm: Nucleus: Eccentricity | Smoothed: 50 µm: Nucleus: Eccentricity |
| Nucleus: Hematoxylin OD mean | Smoothed: 25 µm: Nucleus: Hematoxylin OD mean | Smoothed: 50 µm: Nucleus: Hematoxylin OD mean |
| Nucleus: Hematoxylin OD sum | Smoothed: 25 µm: Nucleus: Hematoxylin OD sum | Smoothed: 50 µm: Nucleus: Hematoxylin OD sum |
| Nucleus: Hematoxylin OD std dev | Smoothed: 25 µm: Nucleus: Hematoxylin OD std dev | Smoothed: 50 µm: Nucleus: Hematoxylin OD std dev |
| Nucleus: Hematoxylin OD max | Smoothed: 25 µm: Nucleus: Hematoxylin OD max | Smoothed: 50 µm: Nucleus: Hematoxylin OD max |
| Nucleus: Hematoxylin OD min | Smoothed: 25 µm: Nucleus: Hematoxylin OD min | Smoothed: 50 µm: Nucleus: Hematoxylin OD min |
| Nucleus: Hematoxylin OD range | Smoothed: 25 µm: Nucleus: Hematoxylin OD range | Smoothed: 50 µm: Nucleus: Hematoxylin OD range |
| Nucleus: Eosin OD mean | Smoothed: 25 µm: Nucleus: Eosin OD mean | Smoothed: 50 µm: Nucleus: Eosin OD mean |
| Nucleus: Eosin OD sum | Smoothed: 25 µm: Nucleus: Eosin OD sum | Smoothed: 50 µm: Nucleus: Eosin OD sum |
| Nucleus: Eosin OD std dev | Smoothed: 25 µm: Nucleus: Eosin OD std dev | Smoothed: 50 µm: Nucleus: Eosin OD std dev |
| Nucleus: Eosin OD max | Smoothed: 25 µm: Nucleus: Eosin OD max | Smoothed: 50 µm: Nucleus: Eosin OD max |
| Nucleus: Eosin OD min | Smoothed: 25 µm: Nucleus: Eosin OD min | Smoothed: 50 µm: Nucleus: Eosin OD min |
| Nucleus: Eosin OD range | Smoothed: 25 µm: Nucleus: Eosin OD range | Smoothed: 50 µm: Nucleus: Eosin OD range |
| Cell: Area | Smoothed: 25 µm: Cell: Area | Smoothed: 50 µm: Cell: Area |
| Cell: Perimeter | Smoothed: 25 µm: Cell: Perimeter | Smoothed: 50 µm: Cell: Perimeter |
| Cell: Circularity | Smoothed: 25 µm: Cell: Circularity | Smoothed: 50 µm: Cell: Circularity |
| Cell: Max caliper | Smoothed: 25 µm: Cell: Max caliper | Smoothed: 50 µm: Cell: Max caliper |
| Cell: Min caliper | Smoothed: 25 µm: Cell: Min caliper | Smoothed: 50 µm: Cell: Min caliper |
| Cell: Eccentricity | Smoothed: 25 µm: Cell: Eccentricity | Smoothed: 50 µm: Cell: Eccentricity |
| Cell: Eosin OD mean | Smoothed: 25 µm: Cell: Eosin OD mean | Smoothed: 50 µm: Cell: Eosin OD mean |
| Cell: Eosin OD std dev | Smoothed: 25 µm: Cell: Eosin OD std dev | Smoothed: 50 µm: Cell: Eosin OD std dev |
| Cell: Eosin OD max | Smoothed: 25 µm: Cell: Eosin OD max | Smoothed: 50 µm: Cell: Eosin OD max |
| Cell: Eosin OD min | Smoothed: 25 µm: Cell: Eosin OD min | Smoothed: 50 µm: Cell: Eosin OD min |
| Cytoplasm: Eosin OD mean | Smoothed: 25 µm: Cytoplasm: Eosin OD mean | Smoothed: 50 µm: Cytoplasm: Eosin OD mean |
| Cytoplasm: Eosin OD std dev | Smoothed: 25 µm: Cytoplasm: Eosin OD std dev | Smoothed: 50 µm: Cytoplasm: Eosin OD std dev |
| Cytoplasm: Eosin OD max | Smoothed: 25 µm: Cytoplasm: Eosin OD max | Smoothed: 50 µm: Cytoplasm: Eosin OD max |
| Cytoplasm: Eosin OD min | Smoothed: 25 µm: Cytoplasm: Eosin OD min | Smoothed: 50 µm: Cytoplasm: Eosin OD min |
| Nucleus/Cell area ratio | Smoothed: 25 µm: Nucleus/Cell area ratio | Smoothed: 50 µm: Nucleus/Cell area ratio |
| | Smoothed: 25 µm: Nearby detection counts | Smoothed: 50 µm: Nearby detection counts |

**Table S2** Cox regression hazard ratios for recurrence-free survival or overall survival and low versus high Ki67 sugroups using DIA hot spot apps, DIA global scoring and manual hot spot scoring

| | Recurrence-free survival | | | | Overall survival | | | |
|---|---|---|---|---|---|---|---|---|
| **Scoring method** | **Hazard ratio** | **95% confidence interval** | | ***p*** | **Hazard ratio** | **95% confidence interval** | | ***p*** |
| | | **lower** | **upper** | | | **lower** | **upper** | |
| APP05 | 3.66 | 1.39 | 9.66 | 0.009* | 6.75 | 1.56 | 29.12 | 0.010* |
| APP06 | 11.06 | 1.50 | 81.50 | 0.018* | 7.57 | 1.01 | 56.64 | 0.049* |
| APP08 | 9.26 | 1.26 | 68.16 | 0.029* | 6.31 | 0.84 | 47.19 | 0.073 |
| APP09 | 5.13 | 1.94 | 13.53 | 0.001* | 5.97 | 1.75 | 20.41 | 0.004* |
| APP10 | 6.83 | 2.36 | 19.74 | <0.001* | 6.07 | 1.78 | 20.74 | 0.004* |
| APP11 | 5.79 | 2.00 | 16.74 | 0.001* | 5.07 | 1.48 | 17.33 | 0.010* |
| APP12 | 5.25 | 1.58 | 17.41 | 0.007* | 3.39 | 0.99 | 11.57 | 0.052 |
| APP13 | 4.15 | 1.44 | 12.00 | 0.008* | 2.66 | 0.89 | 7.96 | 0.081 |
| APP14 | 4.54 | 1.37 | 15.08 | 0.014* | 4.67 | 1.08 | 20.15 | 0.039* |
| APP15 | 5.80 | 1.74 | 19.25 | 0.004* | 5.93 | 1.37 | 25.60 | 0.017* |
| APP16 | 7.68 | 1.04 | 56.57 | 0.045* | 5.24 | 0.70 | 39.19 | 0.107 |
| APP20 | 3.71 | 1.12 | 12.34 | 0.032* | 8.10 | 1.08 | 60.59 | 0.042* |
| APP21 | 4.56 | 1.37 | 15.12 | 0.013* | 9.92 | 1.33 | 74.16 | 0.025* |
| APP22 | 5.19 | 1.56 | 17.22 | 0.007* | 5.31 | 1.23 | 22.90 | 0.025* |
| APP23 | 6.06 | 1.83 | 20.14 | 0.003* | 6.17 | 1.43 | 26.66 | 0.015* |
| **APP24** | **6.88** | **2.07** | **22.87** | **0.002*** | **6.93** | **1.61** | **29.91** | **0.009*** |
| APP25 | 5.19 | 1.80 | 15.02 | 0.002* | 4.53 | 1.33 | 15.48 | 0.016* |
| APP26 | 5.79 | 2.00 | 16.74 | 0.001* | 5.07 | 1.48 | 17.33 | 0.010* |
| APP27 | 4.51 | 1.71 | 11.91 | 0.002* | 8.42 | 1.95 | 36.35 | 0.004* |
| **DIA global** | **3.13** | **1.41** | **6.95** | **0.005*** | **7.46** | **2.46** | **22.58** | **<0.001*** |
| Manual hot spot | 2.76 | 1.16 | 6.53 | 0.021* | 2.27 | 0.87 | 5.92 | 0.093 |

**P* significant at a <0.05 level. DIA, digital image analysis.

**Table S3** Cox regression hazard ratios for recurrence-free survival or overall survival and Ki67 low versus high, luminal A versus B-like surrogate subtypes and PAM50 luminal A versus B subtypes, among pN0 and pN1 cases

| | Recurrence-free survival | | | Overall survival | | |
|---|---|---|---|---|---|---|
| **Scoring method** | **Hazard ratio** | **95% confidence interval** | ***p*** | **Hazard ratio** | **95% confidence interval** | ***p*** |
| pN0 | | | | | | |
| Ki67 DIA hot spot APP24 scoring (<20% vs ≥20%) | 48.80 | 0.20 - 12119.6 | 0.167 | 3.30 | 0.39 - 28.28 | 0.276 |
| Ki67 DIA global scoring (<20% vs >20%) | **4.12** | 1.01 - 16.74 | **0.048*** | 3.57 | 0.42 - 30.62 | 0.245 |
| Surrogate subtype DIA hot spot (lum A vs lum B-like) | 31.79 | 0.05 - 18973.5 | 0.289 | 31.96 | 0.01 - 78122.8 | 0.384 |
| Surrogate subtype DIA global (lum A vs lum B-like) | 1.96 | 0.49 - 7.87 | 0.342 | 70.60 | 0.10 - 50983.9 | 0.205 |
| PAM50 subtype (lum A vs lum B) | 3.34 | 0.83 - 13.37 | 0.089 | 4.92 | 0.82 - 29.46 | 0.081 |
| pN1 | | | | | | |
| Ki67 DIA hot spot APP24 scoring (<20% vs ≥20%) | 6.90 | 0.86 - 55.30 | 0.069 | 6.23 | 0.76 - 50.98 | 0.088 |
| Ki67 DIA global scoring (<20% vs >20%) | 2.74 | 0.68 - 11.06 | 0.158 | 2.54 | 0.51 - 12.62 | 0.254 |
| Surrogate subtype DIA hot spot (lum A vs lum B-like) | 4.97 | 0.62 - 39.82 | 0.131 | 4.31 | 0.53 - 35.05 | 0.172 |
| Surrogate subtype DIA global (lum A vs lum B-like) | 1.81 | 0.45 - 7.23 | 0.402 | 2.81 | 0.57 - 13.94 | 0.207 |
| PAM50 subtype (lum A vs lum B) | **6.57** | 1.24 - 34.87 | **0.027*** | 3.83 | 0.70 - 20.93 | 0.121 |

*$P$ significant at a <0.05 level. DIA, digital image analysis; RFS, recurrence-free survival; OS, overall survival.

**Table S4** Univariate Cox proportional hazard models for recurrence-free survival

| **Variable** | **Hazard ratio** | **95% confidence interval** | | ***p*** |
|---|---|---|---|---|
| | | **lower** | **upper** | |
| pT stage^ | | | | 0.161 |
| pT1 (ref) | - | - | - | 0.165 |
| pT2 | 1.91 | 0.80 | 4.57 | 0.147 |
| pT3 | 3.37 | 0.87 | 13.05 | 0.079 |
| Grade | | | | 0.003* |
| Grade (1 ref) | - | - | - | 0.727 |
| Grade 2 | 27272.91 | <0.001 | 2.43E+65 | 0.887 |
| Grade 3 | 36822.46 | <0.001 | 3.28E+65 | 0.883 |
| Mitotic score | | | | 0.214 |
| Mitotic score (1 ref) | - | - | - | 0.229 |
| Mitotic score 2 | 1.86 | 0.77 | 4.50 | 0.169 |
| Mitotic score 3 | 2.21 | 0.82 | 5.95 | 0.116 |
| pN stage^ | | | | 0.208 |
| pN (pN0 ref) | - | - | - | - |
| pN1 | 1.82 | 0.72 | 4.58 | 0.205 |
| pN stage^ | | | | 0.005* |
| pN (pN0 ref) | - | - | - | 0.002* |
| pN1 | 1.84 | 0.73 | 4.63 | 0.198 |
| pN2 | 4.90 | 1.82 | 13.16 | 0.002* |
| pN3 | 7.05 | 1.91 | 26.10 | 0.003* |

*$P$ significant at a <0.05 level. ^Pathologic T stage (pT) for invasive tumor and pathologic N stage (pN) for regional lymph nodes according to AJCC Breast Cancer Staging 7th Edition (TNM 7).

**Table S5** Multivariate Cox proportional hazard models for recurrence-free survival

| Variables in model | Hazard ratio | 95% confidence interval | *p* |
|---|---|---|---|
| pN stage^ (pN0 ref) | - | - | - |
| pN1 | 1.98 | 0.79 - 4.99 | 0.148 |
| pN2 | 5.22 | 1.94 - 14.06 | 0.001* |
| pN3 | 12.39 | 3.27 - 46.92 | <0.001* |
| Ki67 DIA hot spot APP24 scoring (≥20% vs <20%) | 8.42 | 2.49 - 28.45 | 0.001* |
| pN stage (pN0 ref) | - | - | - |
| pN1 | 1.54 | 0.61 - 3.89 | 0.365 |
| pN2 | 4.27 | 1.52 - 12.02 | 0.006* |
| pN3 | 5.02 | 1.08 - 23.33 | 0.040* |
| Ki67 DIA global scoring (≥20% vs <20%) | 3.23 | 1.45 - 7.20 | 0.004* |
| pN stage (pN0 ref) | - | - | - |
| pN1 | 1.44 | 0.57 - 3.65 | 0.436 |
| pN2 | 4.15 | 1.47 - 11.73 | 0.007* |
| pN3 | 6.95 | 1.87 - 25.87 | 0.004* |
| Ki67 manual hot spot scoring (≥20% vs <20%) | 2.78 | 1.16 - 6.65 | 0.022* |

**P* significant at a <0.05 level. ^Pathologic N stage for regional lymph nodes according to AJCC Breast Cancer Staging 7th Edition (TNM 7). DIA, digital image analysis.

**Table S6** Univariate Cox proportional hazard models for overall survival

| Variable | Hazard ratio | 95% confidence interval | | *p* |
|---|---|---|---|---|
| | | lower | upper | |
| pT stage^ | | | | 0.161 |
| pT1 (ref) | - | - | - | 0.199 |
| pT2 | 2.45 | 0.81 | 7.43 | 0.115 |
| pT3 | 3.81 | 0.70 | 20.79 | 0.123 |
| Grade | | | | <0.001* |
| Grade (1 ref) | - | - | - | 0.001 |
| Grade 2 | 1.59 | 0.19 | 13.62 | 0.672 |
| Grade 3 | 8.98 | 1.18 | 68.45 | 0.034 |
| Mitotic score | | | | 0.009* |
| Mitotic score (1 ref) | - | - | - | 0.011 |
| Mitotic score 2 | 2.56 | 0.75 | 8.75 | 0.134 |
| Mitotic score 3 | 6.11 | 1.83 | 20.38 | 0.003 |
| pN stage^ | | | | 0.112 |
| pN (pN0 ref) | - | - | - | - |
| pN1 | 2.35 | 0.81 | 6.77 | 0.114 |
| pN stage^ | | | | 0.066 |
| pN (pN0 ref) | - | - | - | 0.061 |
| pN1 | 2.37 | 0.82 | 6.82 | 0.111 |
| pN2 | 4.04 | 1.14 | 14.33 | 0.030 |
| pN3 | 6.19 | 1.25 | 30.76 | 0.026 |

**P* significant at a <0.05 level. ^Pathologic T stage (pT) for invasive tumor and pathologic N stage (pN) for regional lymph nodes according to AJCC Breast Cancer Staging 7th Edition (TNM 7).

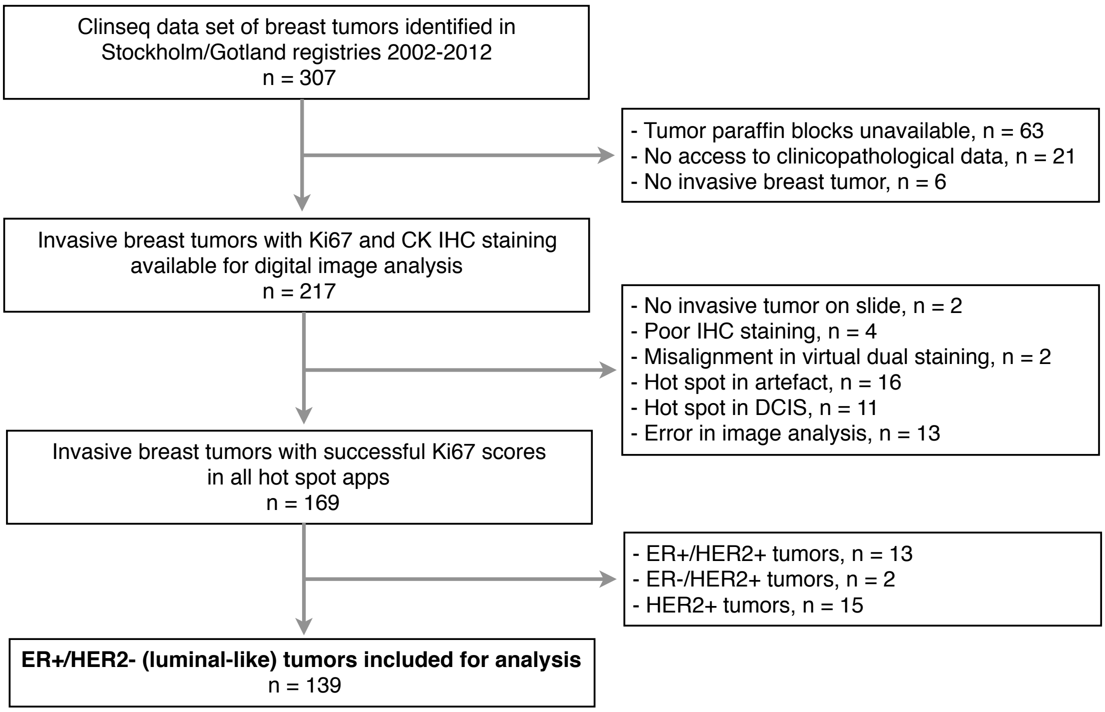


**Fig. S1** CONSORT diagram of the study cohort. CK, cytokeratin; IHC, immunohistochemistry; DCIS, ductal carcinoma *in situ*; ER, estrogen receptor; HER2, human epidermal growth factor receptor 2.

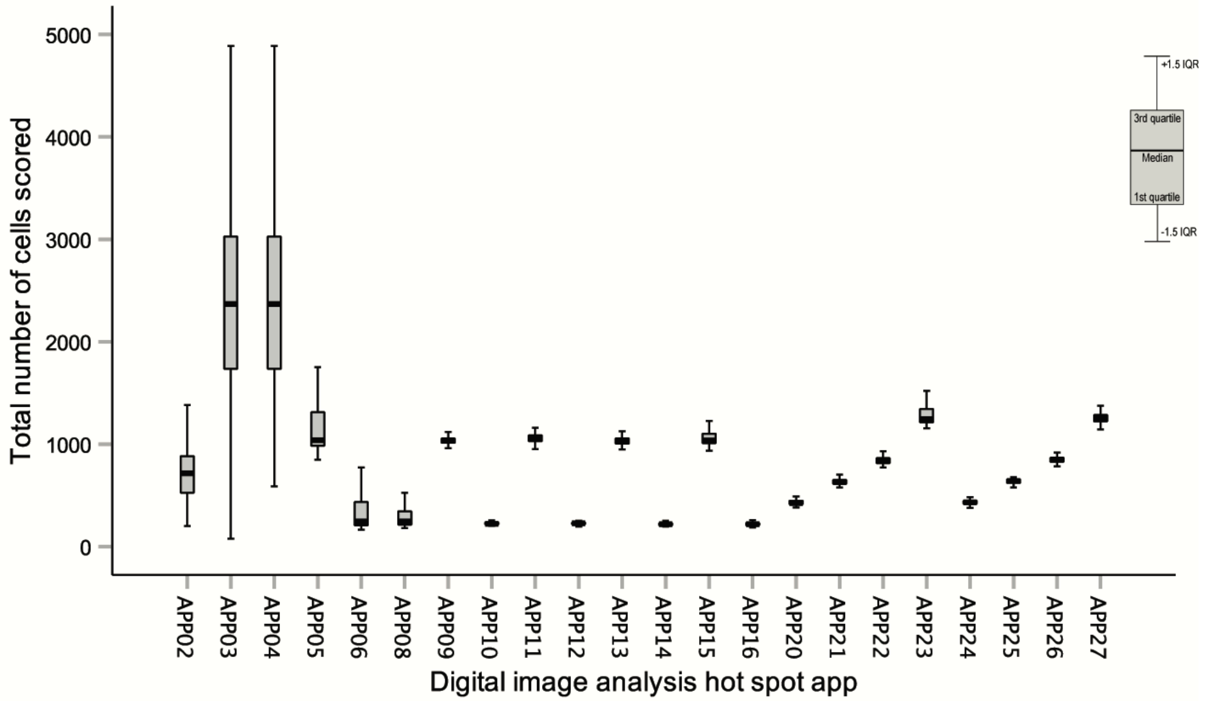


**Fig. S2** Distribution of the total number of cells scored for each automated hot spot app. IQR, interquartile range.

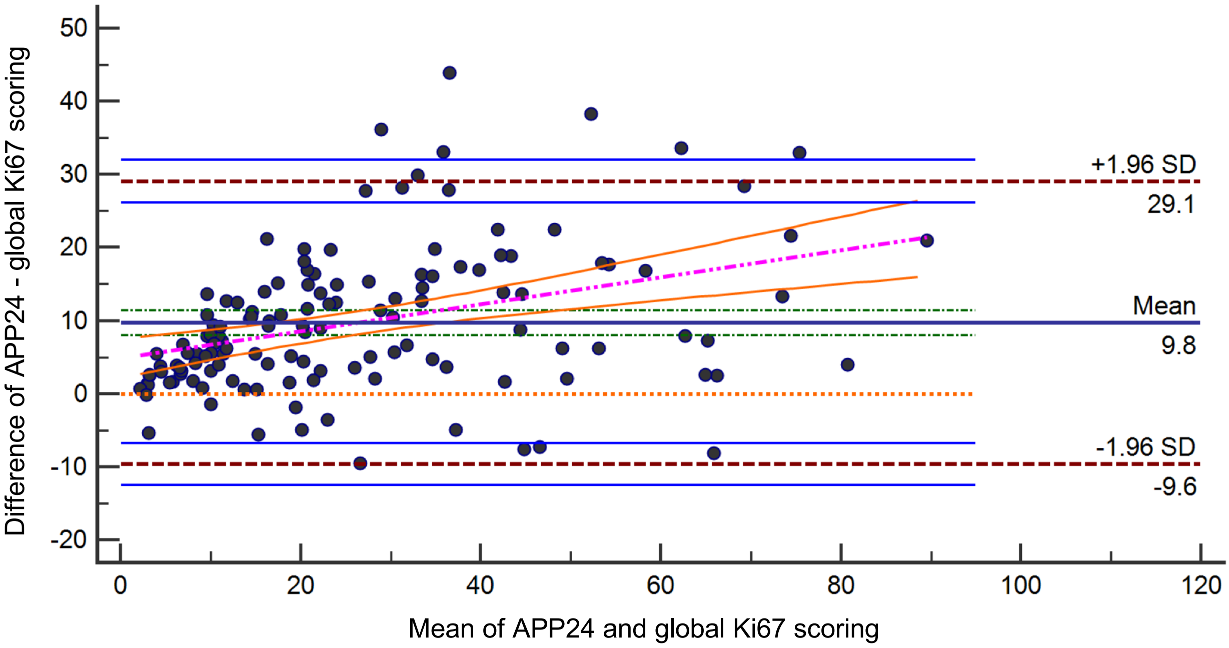


**Fig. S3** Bland-Altman plot illustrating the difference between DIA hot spot APP24 and DIA global Ki67 values plotted against the mean Ki67 values of the paired DIA methods (mean Ki67 of DIA hot spot APP24 and DIA global Ki67 calculated for each case). The expected mean difference is 0, shown by dashed orange line. The observed mean difference is shown as a horizontal dark blue line and the limits of agreement as dashed brown lines. The regression line is shown in pink. DIA, digital image analysis. The 95% confidence intervals are shown for each metric.

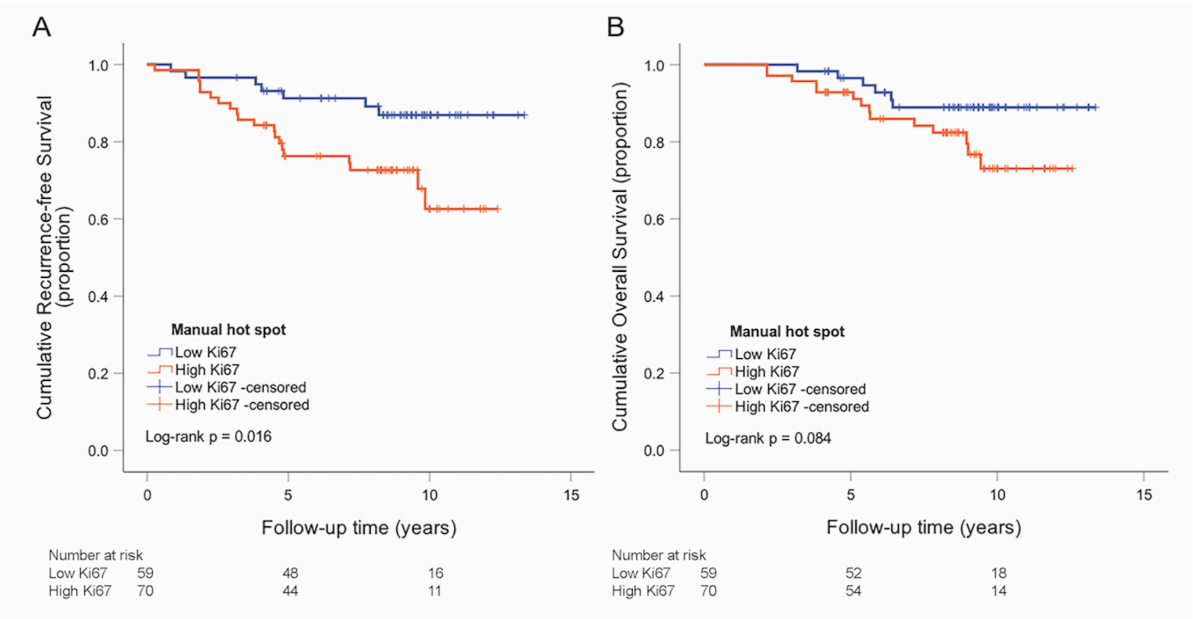


**Fig. S4** Kaplan-Meier curves demonstrating associations between low versus high Ki67 subgroups and recurrence-free survival (a) or overall survival (b) using manual hot spot scoring.

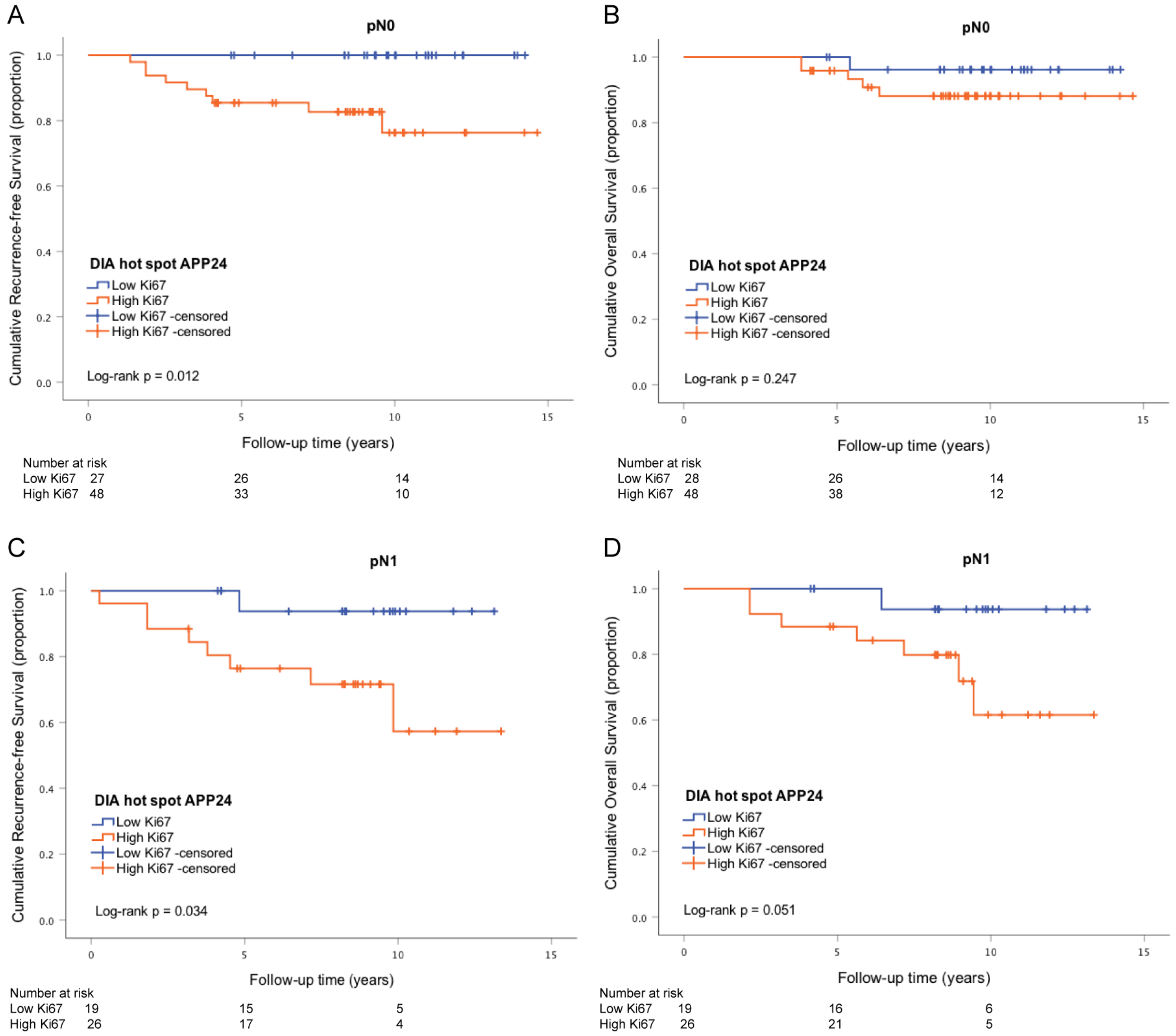


**Fig. S5** Kaplan-Meier curves demonstrating associations between low versus high Ki67 subgroups and recurrence-free survival or overall survival in pN0 cases (a-b) and pN1 cases (c-d) using DIA hot spot APP24 scoring.

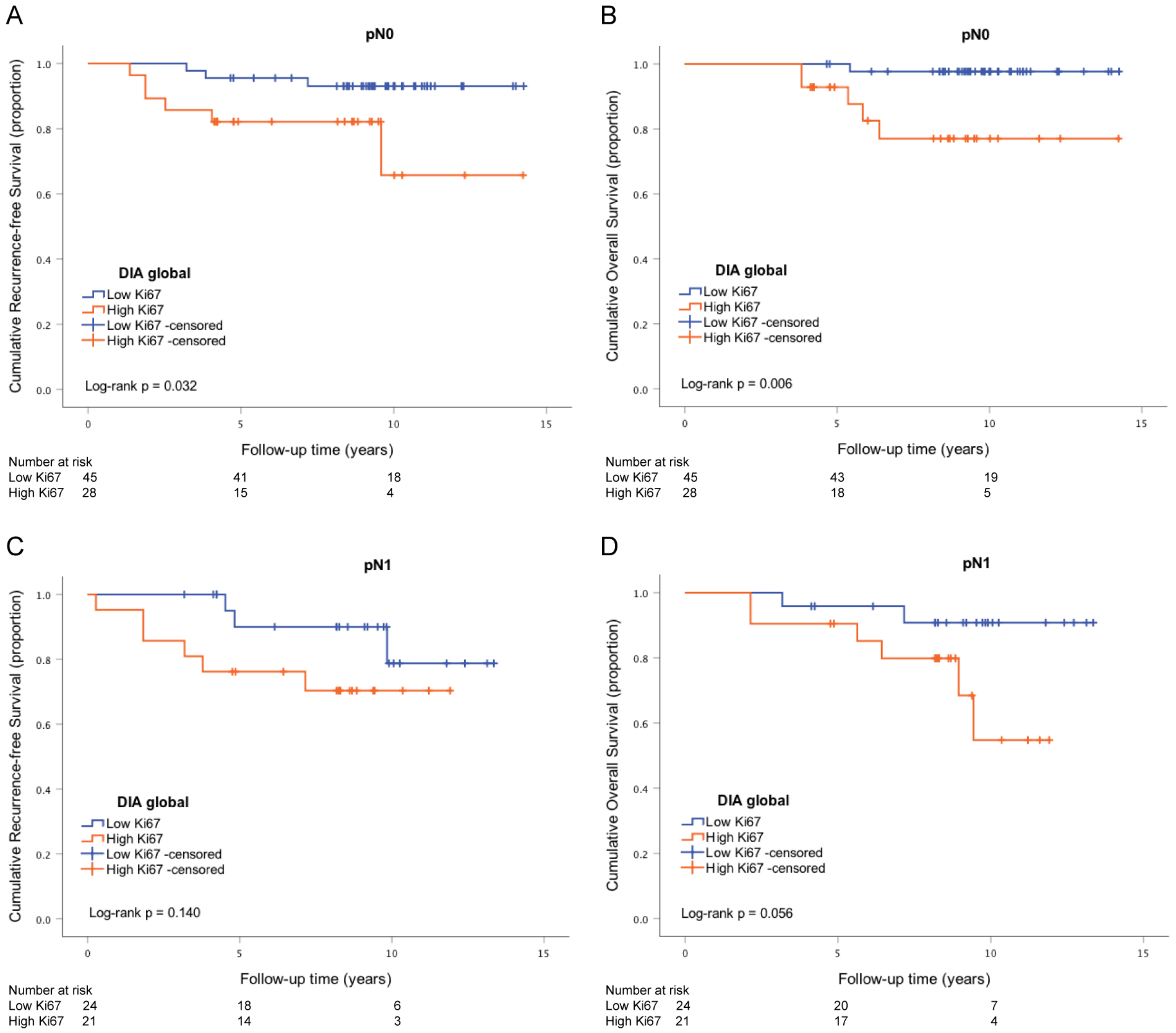


**Fig. S6** Kaplan-Meier curves demonstrating associations between low versus high Ki67 subgroups and recurrence-free survival or overall survival in pN0 cases (a-b) and pN1 cases (c-d) using DIA global scoring.

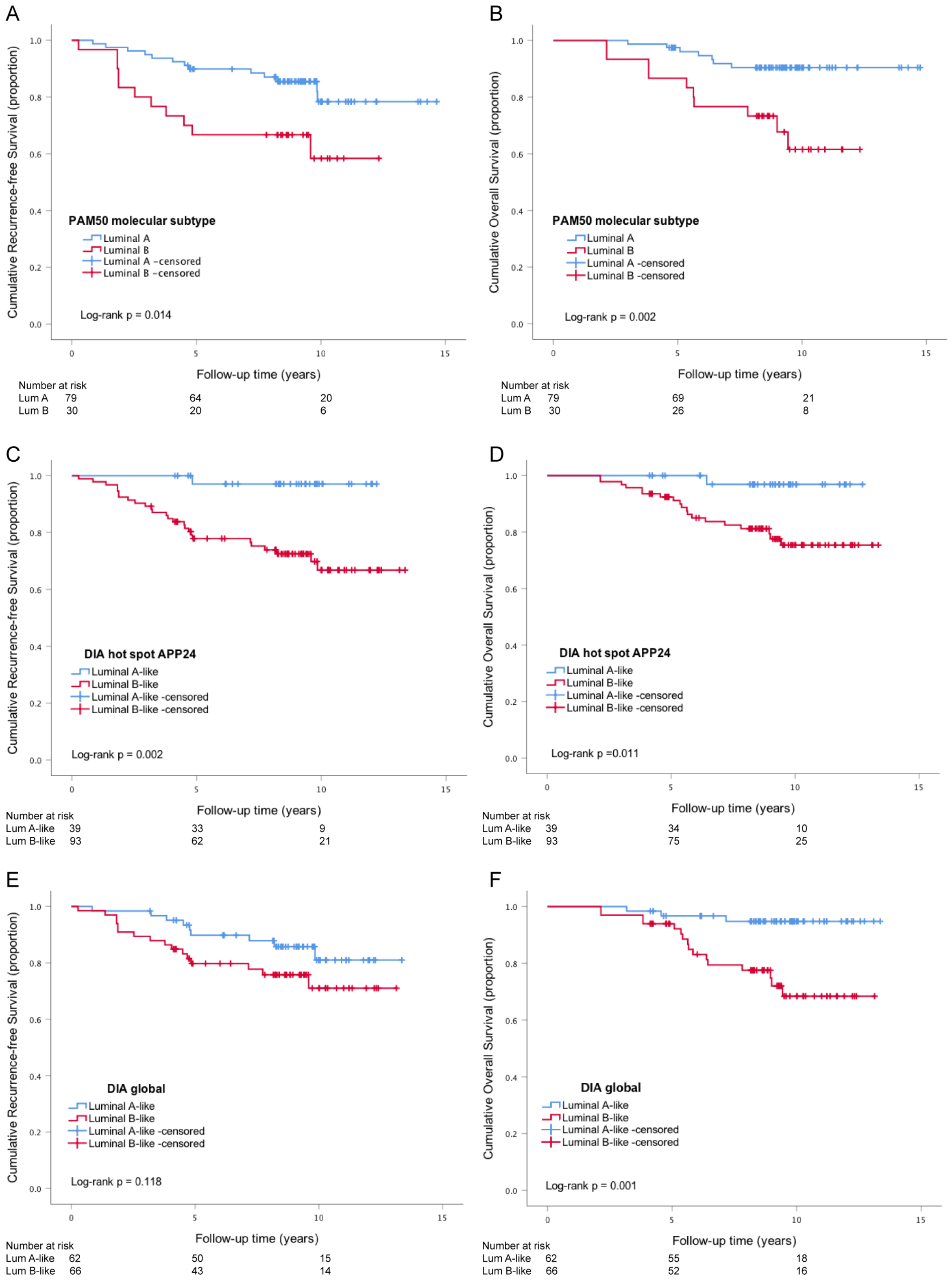


**Fig. S7** Kaplan-Meier curves demonstrating associations between luminal A versus luminal B tumors and recurrence-free (a) or overall survival (b) determined by PAM50 molecular intrinsic subtypes. Associations between luminal A-like versus luminal B-like tumors and recurrence-free (c) or overall survival (d) using DIA hot spot APP24 scoring. Associations between luminal A-like versus luminal B-like tumors and recurrence-free (e) or overall survival (f) using DIA global scoring.
